# Supplementary material for: Comparative Effectiveness of Treatments for Shoulder Subluxation After Stroke: A Systematic Review and Network Meta-Analysis
Source: J Clin Med. 2025 Sep 29;14(19):6913. doi: 10.3390/jcm14196913 (PMC12525310; doi:10.3390/jcm14196913)
Supplement: Supplementary file 1 [file jcm-14-06913-s001.zip › jcm-3871253-supplementary.pdf]

Supplementary Material

Table S1. PRISMA NMA Checklist

Table S2. Search strategy

Table S3. Main outcomes of the studies included in this systematic review and meta-analysis

Figure S1. Risk of bias for randomized controlled trials assessed using the Cochrane Risk of Bias 2.0 Tool.

Figure S2. Funnel plot to detect publication bias

Table S4A. Results of local inconsistency tests (node-splitting analysis)

Table S4B. Global inconsistency assessment (design-by-treatment interaction model).

Table S5 SUCRA Values of Treatments for Shoulder Subluxation in Stroke Patients.

Table S6 Indirectness for all studies

Table S7 Confidence in Network Meta-analysis (CINeMA) final report

Table S1. **RISMA NMA Checklist of Items to Include When Reporting A Systematic Review Involving a Network Meta-analysis**

Section/Topic	Item #	Checklist Item	Reported on Page #
**TITLE**			
Title	1	Identify the report as a systematic review *incorporating a network meta-analysis (or related form of meta-analysis).*	**1**
**ABSTRACT**			
Structured summary	2	Provide a structured summary including, as applicable:<br>**Background:** main objectives<br>**Methods:** data sources; study eligibility criteria, participants, and interventions; study appraisal; and *synthesis methods, such as network meta-analysis.*<br>**Results:** number of studies and participants identified; summary estimates with corresponding confidence/credible intervals; *treatment rankings may also be discussed. Authors may choose to summarize pairwise comparisons against a chosen treatment included in their analyses for brevity.*<br>**Discussion/Conclusions:** limitations; conclusions and implications of findings.<br>**Other:** primary source of funding; systematic review registration number with registry name.	**1**
**INTRODUCTION**			
Rationale	3	Describe the rationale for the review in the context of what is already known*, including mention of why a network meta-analysis has been conducted.*_	**2**
Objectives	4	Provide an explicit statement of questions being addressed, with reference to participants, interventions, comparisons, outcomes, and study design (PICOS).	**2**
**METHODS**			
Protocol and registration	5	Indicate whether a review protocol exists and if and where it can be accessed (e.g., Web address); and, if available, provide registration information, including registration number.	**3**
Eligibility criteria	6	Specify study characteristics (e.g., PICOS, length of follow-up) and report characteristics (e.g., years considered, language, publication status) used as criteria for eligibility, giving rationale. *Clearly describe eligible treatments included in the treatment network, and note whether any have been clustered or merged into the same node (with justification).*_	**3**

Information sources	7	Describe all information sources (e.g., databases with dates of coverage, contact with study authors to identify additional studies) in the search and date last searched.	**3**
Search	8	Present full electronic search strategy for at least one database, including any limits used, such that it could be repeated.	**3**
Study selection	9	State the process for selecting studies (i.e., screening, eligibility, included in systematic review, and, if applicable, included in the meta-analysis).	**3**
Data collection process	10	Describe method of data extraction from reports (e.g., piloted forms, independently, in duplicate) and any processes for obtaining and confirming data from investigators.	**3-4**
Data items	11	List and define all variables for which data were sought (e.g., PICOS, funding sources) and any assumptions and simplifications made.	**3-4**
**Geometry of the network**	**S1**	Describe methods used to explore the geometry of the treatment network under study and potential biases related to it. This should include how the evidence base has been graphically summarized for presentation, and what characteristics were compiled and used to describe the evidence base to readers.	**Fig 2**
Risk of bias within individual studies	12	Describe methods used for assessing risk of bias of individual studies (including specification of whether this was done at the study or outcome level), and how this information is to be used in any data synthesis.	**3**
Summary measures	13	State the principal summary measures (e.g., risk ratio, difference in means). *Also describe the use of additional summary measures assessed, such as treatment rankings and surface under the cumulative ranking curve (SUCRA) values, as well as modified approaches used to present summary findings from meta-analyses.*	**3-4**
Planned methods of analysis	14	Describe the methods of handling data and combining results of studies for each network meta-analysis. This should include, but not be limited to:<br>• *Handling of multi-arm trials;*<br>• *Selection of variance structure;*<br>• *Selection of prior distributions in Bayesian analyses; and*<br>• *Assessment of model fit.*	**3-4**
**Assessment of Inconsistency**	**S2**	Describe the statistical methods used to evaluate the agreement of direct and indirect evidence in the treatment network(s) studied. Describe efforts taken to address its presence when found.	**4**
Risk of bias across studies	15	Specify any assessment of risk of bias that may affect the cumulative evidence (e.g., publication bias, selective reporting within studies).	**FigS1**
Additional analyses	16	Describe methods of additional analyses if done, indicating which were pre-specified. This may include, but not be limited to, the following:<br>• Sensitivity or subgroup analyses;	**N/A-**

- Meta-regression analyses;
- *Alternative formulations of the treatment network; and*
- *Use of alternative prior distributions for Bayesian analyses (if applicable).*_

RESULTS†

Study selection	17	Give numbers of studies screened, assessed for eligibility, and included in the review, with reasons for exclusions at each stage, ideally with a flow diagram.	**4**
**Presentation of network structure**	**S3**	Provide a network graph of the included studies to enable visualization of the geometry of the treatment network.	**Fig2**
**Summary of network geometry**	**S4**	Provide a brief overview of characteristics of the treatment network. This may include commentary on the abundance of trials and randomized patients for the different interventions and pairwise comparisons in the network, gaps of evidence in the treatment network, and potential biases reflected by the network structure.	**4**
Study characteristics	18	For each study, present characteristics for which data were extracted (e.g., study size, PICOS, follow-up period) and provide the citations.	**Table S3**
Risk of bias within studies	19	Present data on risk of bias of each study and, if available, any outcome level assessment.	**Fig S2**
Results of individual studies	20	For all outcomes considered (benefits or harms), present, for each study: 1) simple summary data for each intervention group, and 2) effect estimates and confidence intervals. *Modified approaches may be needed to deal with information from larger networks.*	**Table S3**
Synthesis of results	21	Present results of each meta-analysis done, including confidence/credible intervals. *In larger networks, authors may focus on comparisons versus a particular comparator (e.g. placebo or standard care), with full findings presented in an appendix. League tables and forest plots may be considered to summarize pairwise comparisons.* If additional summary measures were explored (such as treatment rankings), these should also be presented.	**6-10**
**Exploration for inconsistency**	**S5**	Describe results from investigations of inconsistency. This may include such information as measures of model fit to compare consistency and inconsistency models, *P* values from statistical tests, or summary of inconsistency estimates from different parts of the treatment network.	**5**
Risk of bias across studies	22	Present results of any assessment of risk of bias across studies for the evidence base being studied.	**FigS2**
Results of additional analyses	23	Give results of additional analyses, if done (e.g., sensitivity or subgroup analyses, meta-regression analyses*, alternative network geometries studied, alternative choice of prior distributions for Bayesian analyses,* and so forth).	**N/A**

**DISCUSSION**			
Summary of evidence	24	Summarize the main findings, including the strength of evidence for each main outcome; consider their relevance to key groups (e.g., healthcare providers, users, and policy-makers).	**10-11**
Limitations	25	Discuss limitations at study and outcome level (e.g., risk of bias), and at review level (e.g., incomplete retrieval of identified research, reporting bias). *Comment on the validity of the assumptions, such as transitivity and consistency. Comment on any concerns regarding network geometry (e.g., avoidance of certain comparisons).*	**12-13**
Conclusions	26	Provide a general interpretation of the results in the context of other evidence, and implications for future research.	**13**
**FUNDING**			
Funding	27	Describe sources of funding for the systematic review and other support (e.g., supply of data); role of funders for the systematic review. This should also include information regarding whether funding has been received from manufacturers of treatments in the network and/or whether some of the authors are content experts with professional conflicts of interest that could affect use of treatments in the network.	**13**

PICOS = population, intervention, comparators, outcomes, study design.
* Text in italics indicateS wording specific to reporting of network meta-analyses that has been added to guidance from the PRISMA statement.
† Authors may wish to plan for use of appendices to present all relevant information in full detail for items in this section.

Table S2 Search strategy

Search date: 2024.7.5 > Updated 2025.8.8

Database	Search strategy	Results
**Pubmed**	("Stroke"[Mesh] OR infarction OR intracranial hemorrhage OR hemiplegia OR cerebral vascular accident) AND ("Shoulder Dislocation"[Mesh] OR "shoulder subluxation" OR "glenohumeral subluxation" OR "glenohumeral dislocation" OR "shoulder pain") AND ("Electric Stimulation"[Mesh] OR "electrical stimulation" OR "functional electrical stimulation" OR sling OR "orthotic devices" OR orthoses OR orthosis OR taping OR electroacupuncture OR botox) AND (randomizedcontrolledtrial[Filter])	**60**
**EMBASE**	(('cerebrovascular accident'/exp OR infarction OR intracranial) AND hemorrhage OR hemiplegia OR cerebral) AND ('Electric Stimulation'/exp OR 'electrical stimulation' OR 'functional electrical stimulation' OR sling OR 'orthotic devices' OR orthoses OR orthosis OR taping OR electroacupuncture OR botox) AND ('Shoulder Dislocation'/exp OR 'shoulder subluxation' OR 'glenohumeral subluxation' OR 'glenohumeral dislocation' OR 'shoulder pain') AND 'randomized controlled'	**92**
**Cochran**	Search Name:<br>Date Run: 08/08/2025 09:54:02<br>Comment:<br>ID Search Hits<br>#1 MeSH descriptor: [Stroke] explode all trees 18293	**111**

	#2 MeSH descriptor: [Shoulder Dislocation] explode all trees 247<br>#3 MeSH descriptor: [Electric Stimulation] explode all trees 2600<br>#4 (#1 OR infarction OR intracranial hemorrhage OR hemiplegia OR cerebral vascular accident) AND (#2 OR shoulder subluxation OR glenohumeral subluxation OR glenohumeral dislocation OR shoulder pain) 493<br>#5 #4 AND (#3 OR electrical stimulation OR functional electrical stimulation OR sling OR orthotic devices OR orthoses OR orthosis OR taping OR electroacupuncture OR botox) 160<br>#6 randomized controlled trial 1161804<br>#7 #5 AND #6 111	
**Scopus**	(Stroke OR infarction OR intracranial hemorrhage OR hemiplegia OR cerebral vascular accident) AND ("Shoulder Dislocation" OR "shoulder subluxation" OR "glenohumeral subluxation" OR "glenohumeral dislocation" OR "shoulder pain") AND ("Electric Stimulation" OR "electrical stimulation" OR "functional electrical stimulation" OR sling OR "orthotic devices" OR orthoses OR orthosis OR taping OR electroacupuncture OR botox) AND ("randomized controlled trial")	**156**
**Web of Science**	(Stroke OR infarction OR intracranial hemorrhage OR hemiplegia OR cerebral vascular accident) AND ("Shoulder Dislocation" OR "shoulder subluxation" OR "glenohumeral subluxation" OR "glenohumeral dislocation" OR "shoulder pain") AND ("Electric Stimulation" OR "electrical stimulation" OR "functional electrical	**64**

	stimulation" OR sling OR "orthotic devices" OR orthoses OR orthosis OR taping OR electroacupuncture OR botox) AND ("randomized controlled trial")	

Table S3 Main outcomes of the studies included in this systematic review and meta-analysis

Author (Year)	Study design	Stroke onset duration (months) (Mean ± SD) (Experimental/Control groups)	Experimental group (n)	Control group (n)	Age (Mean ± SD) (Experimental/ Control groups)	Female (%)	Follow-up duration	Outcome measure	Main finding	Adverse effects
Anke van Bladel (2017)	RCT	2.36 ± 1.34/ 2.11 ± 1.01	Sling (n=9)	Standard treatment (n=9)	62 ± 12/ 56 ± 9	39.3	0, 6 wks	1. AHD<br>2. VAS<br>3. Pain questionnaire<br>4. PROM<br>5. MAS<br>6. TIS<br>7. FMA	Not wearing a sling is related to AHD reduction, whereas wearing the sling does not seem to prevent pain and shoulder subluxation. Shoulderlift tends to provide better initial correction than the Actimove sling. However, both slings seem to lose their corrective functioning ability over time.	Not reported
Canan Turkkan (2017)	RCT	4.0 ± 3.3/ 3.7 ± 2.6	NMES (n=12)	Standard treatment (n=12)	61.5 ± 10.4 / 66.7 ± 18.1	58.3	0, 4 wks	1. BMRS<br>2. SDQ<br>3. VAS<br>4. AGT distance<br>5. Thickness of the supraspinatus, upper trapezius, posterior deltoid	SDQ, acromion-greater tuberosity distance, and supraspinatus muscle thickness were improved in the NMES group compared to the control group. The VAS-pain scores decreased in both groups. There was no statistically significant alterations in the other measurements in both groups.	Not reported
Chen Lavi (2022)	double-blind RCT	0.50 ± 0.97/ 1.38 ± 1.61	NMES (n=10)	Sling (n=13)	73.30 ± 9.81 / 67.54 ± 15.54	39.1	0, 6, 8 wks	1. Shoulder subluxation<br>2. FMA<br>3. NPRS<br>4. FIM	Subluxation measured in centimeters was significantly lower in the experimental group than in the control group. No significant group difference in shoulder NPRS was observed in the post-intervention and follow-up measurements.	None
Engin Koyuncu (2010)	RCT	Median 6 (1–48)/ 3 (1.5–8)	NMES (n=25)	Standard treatment (n=25)	60.7 ± 9.49 / 62.0 ± 9.72	72	0, 4 wks	1. VAS<br>2. PROM<br>3. AROM<br>4. Shoulder subluxation stage<br>5. Shoulder subluxation distance	Applying FES treatment to the supraspinatus and posterior deltoid muscles in addition to conventional treatment when treating the subluxation in hemiplegic patients is more beneficial than conventional treatment by itself.	Not reported
JongEun Yim (2024)	double-blind RCT	14.61 ± 5.78/ 15.12 ± 5.59	Kinesio taping (n=18)	Sham taping + standard treatment (n=17)	62.56 ± 4.16 / 61.41 ± 4.43	28.6	0, 6 wks	1. Shoulder subluxation distance<br>2. AROM<br>3. VAS<br>4. SPADI<br>5. Modified BI	Kinesio taping is significantly effective in improving SSD, AROM, VAS, SPADI, and MBI in patients with hemiplegic shoulder subluxation.	Not reported
Kenta Fujimura (2024)	RCT	1.13 ± 0.77/ 1.36 ± 0.67	rPMS (n=22)	Standard treatment (n=24)	69 ±13 / 61 ± 15	32.6	0, 6, 12 wks	1. AHI<br>2. FMA<br>3. MAS<br>4. NRS<br>5. AROM of shoulder abduction	The change in AHI was significantly lower in the rPMS group than in the control group. Moreover, AHI did not show differences in patients with severe impairment but decreased in the rPMS group in patients with mild impairment.	None
Lakse E (2009)	RCT	Median 11.7(10.3-22)/ 12.3 (7.2–64)	Local steroid injection (intra-articular or subacromial space) (n=21)	Standard treatment (n=17)	62.2 ± 9.1 / 66.3 ± 6.7	52.6	0, 1, 4 wks	1. Range of motion<br>2. VAS<br>3. Brunnstrom upper extremity staging<br>4. MAS	In both groups, shoulder range of motion and shoulder pain scores showed significant improvement. When the two groups were compared, the improvement was more significant in the injection group.	None

Lin Yang (2018)	double-blind RCT	4.58 ± 0.21/ 4.80 ± 0.62	Kinesio taping (n=10)	Sham taping + standard treatment (n=9)	59 ± 3.2 / 60 ± 2.3	31.6	0, 1 day 4 wks	1. NPRS 2. Shoulder subluxation 3. AROM 4. EMG (average amplitude)	Kinesio taping is effective in reducing the shoulder pain and subluxation as well as increasing muscle activity and AROM in patients with HSP after stroke.	None
Minghong Sui (2021)	Single blind RCT	Median 1.1 (0.8–3.8)/ 1.46 (0.83–3.73)	Electroacupuncture (n=17)	Sham + standard treatment (n=15)	51 ± 12.44 / 54.4 ± 8.16	31.3	0, 2 wks	1. AHD 2. AGT 3. ALT 4. VAS	Pain and shoulder subluxation showed significantly effective within-group difference in both groups. The between-group difference appeared in the pain intensity, while it disappeared in the three measures of shoulder subluxation.	Not reported
Ozgur Z. Karaahmet (2018)	RCT	1.56 ± 0.34/ 1.17 ± 1.19	NMES (n=12)	Standard treatment (n=9)	56 ± 17.5 / 58 ± 15.4	38.1	0, 4 wks	1. AHD 2. NRS 3. Brunnstrom scale 4. FMA 5. FAT 6. FIM	Combining FES-cycling with a standard rehabilitation program alleviates shoulder pain and may prevent the development of shoulder subluxation over time.	None
Pouran D. Faghri (1994)	RCT	0.56 ± 0.13/ 0.53 ± 0.16	NMES (n=13)	Standard treatment (n=13)	65 ± 13 / 69 ± 12	42.3	0, 6, 12 wks	1. Modified Bobath assessment chart 2. SROMP 3. Modified gross clinical scale 4. Surface EMG activity 5. Anteroposterior x-ray (differences between the involved and uninvolved upper extremities)	The experimental group showed significant improvements in arm function, electromyographic activity of the posterior deltoid, and range of motion, and reduction in subluxation (as indicated by x-ray) compared to the control group.	Not reported
Sandra L. Linn (1999)	Single blind RCT	Within 0.07	NMES (n=20)	Standard treatment (n=20)	71 / 73	55	0, 4, 12 wks	1. Subluxation grade 2. Pain-free range of PLRL 3. Verbal rating scale 4. Motor assessment scale 5. Upper arm girth	The treatment group had significantly less subluxation and pain after the treatment period; however, at the end of the follow-up period. there were no significant differences between the two groups.	Not reported
Subhasish Chatterjee (2016)	RCT	0.71± 0.09/ 0.82 ± 0.25	Kinesio taping (n=15)	Standard treatment (n=15)	63.2 ± 4.0 / 62.8 ± 4.5	43.3	0, 6 wks	1. AHD 2. VAS 3. Shoulder flexion AROM 4. FMA	The taping method demonstrated a significant reduction of pain in the treatment group from baseline, a significant improvement in active shoulder flexion, and a significant improvement in proximal arm function. Shoulder subluxation was not statistically significant.	Not reported

SD, Standard deviation; RCT, randomized controlled trial; AHD, Acromio-humeral distance; VAS, visual analogue scale; PROM, passive range of motion; MAS, Modified Ashworth Scale; TIS, trunk impairment scale; FMA, Fugl-Meyer assessment; NMES, neuromuscular electrical stimulation; BMRS, Brunnstrom motor recovery stage; SDQ, shoulder disability questionnaire; AGT, Acromion-Greater Tuberosity; NPRS, numerical pain rating scale; FIM, functional independence measure; AROM, active range of motion; SPADI, shoulder pain and disability index; BI, Barthel index; rPMS, repetitive peripheral magnetic stimulation; AHI, Acromio-humeral interval; NRS, numerical rating scale; EMG, electromyography; ALT, Acromion-Lesser Tuberosity; FAT, Frenchay arm test; SROMP, shoulder range of movement at the point of pain; PLRL, passive lateral rotation.

Figure S1. Risk of bias for randomized controlled trials assessed using the Cochrane Risk of Bias 2.0 Tool.

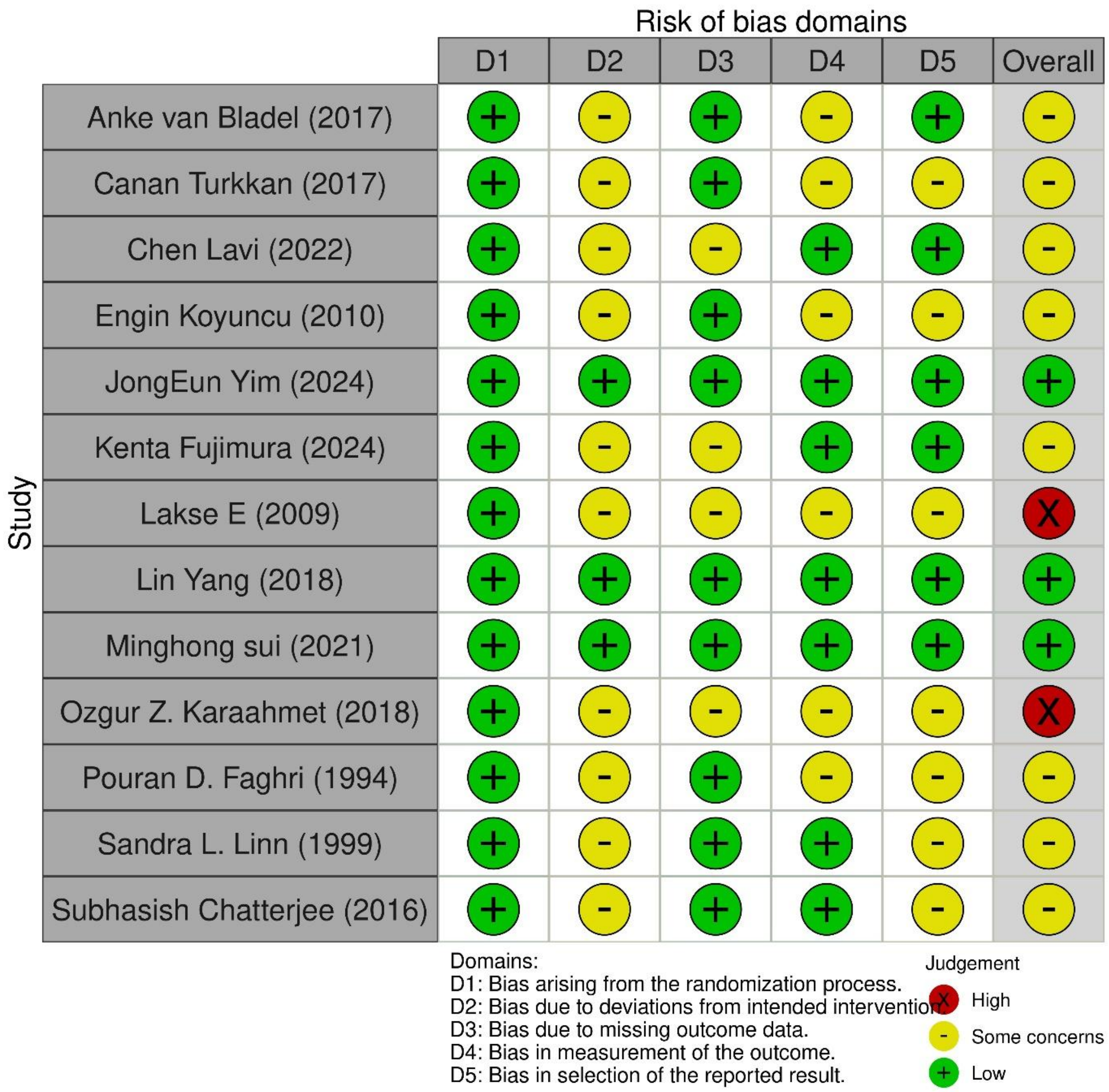

Figure S2. Funnel plot to detect publication bias

(A) Shoulder Subluxation Distance

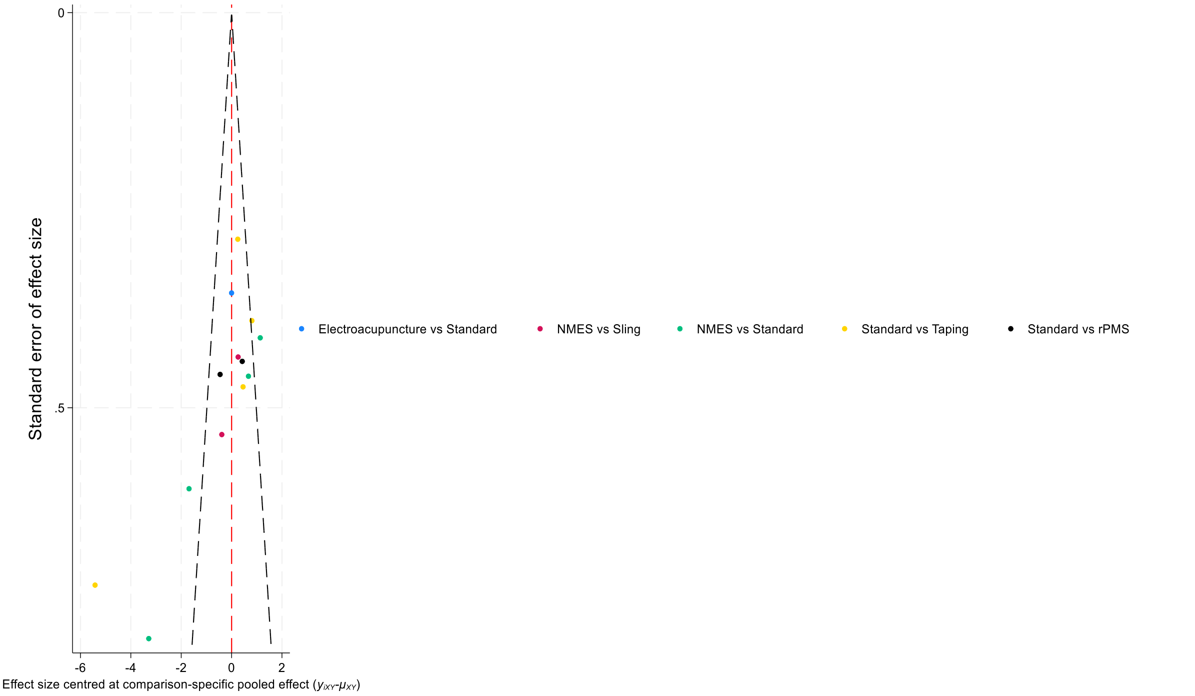

Egger's test P = 0.006

(B) Pain at Rest

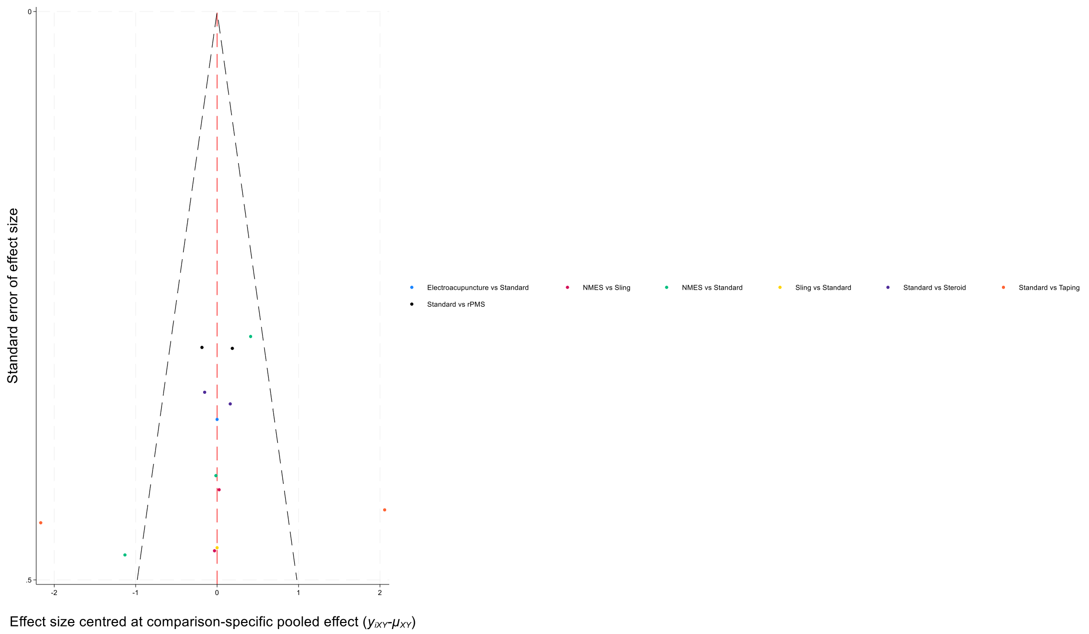

Egger's test P = 0.474

(C) Pain during Activity

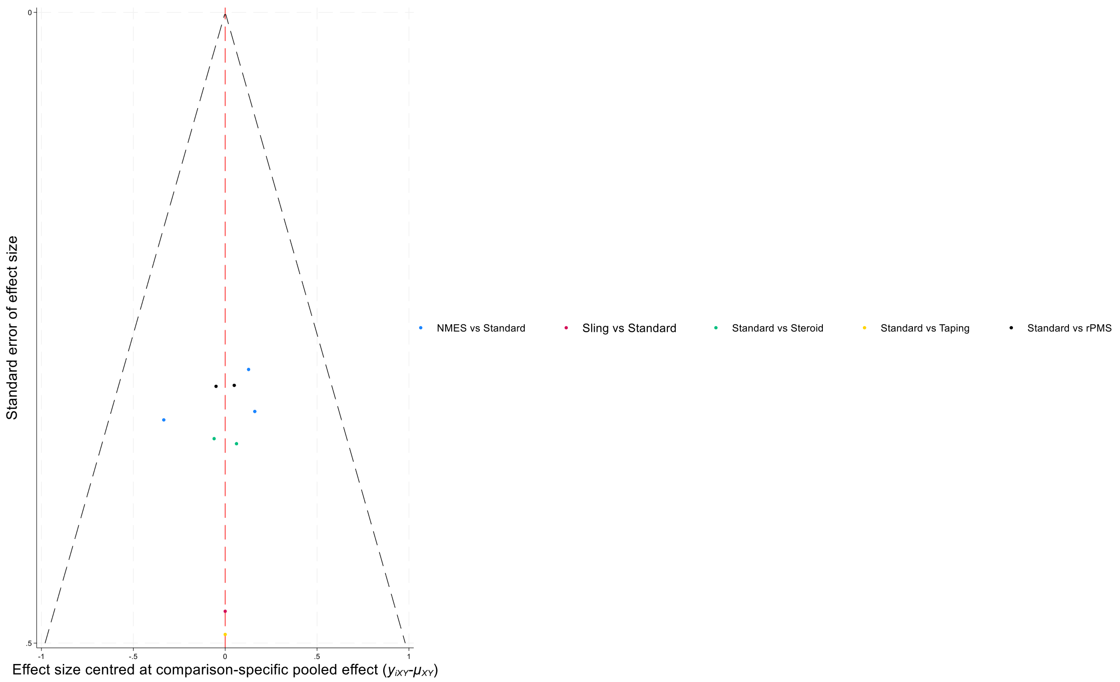

Egger's test P = 0.777

(D) Fugl-Meyer Assessment

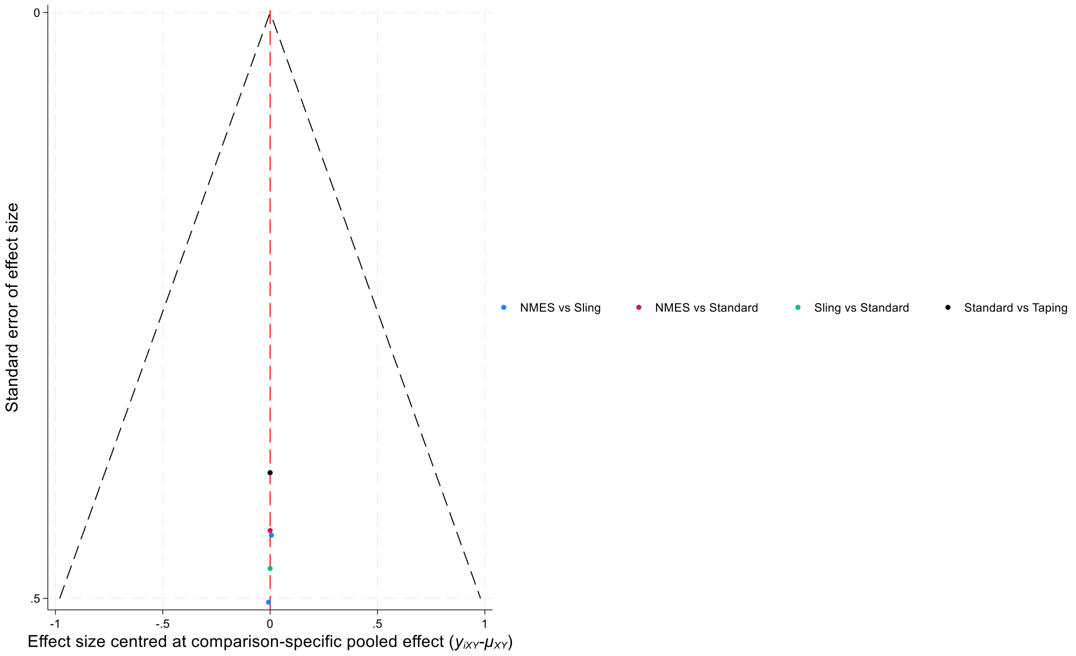

Egger's test P = 0.442

(E) PROM Shoulder Abduction

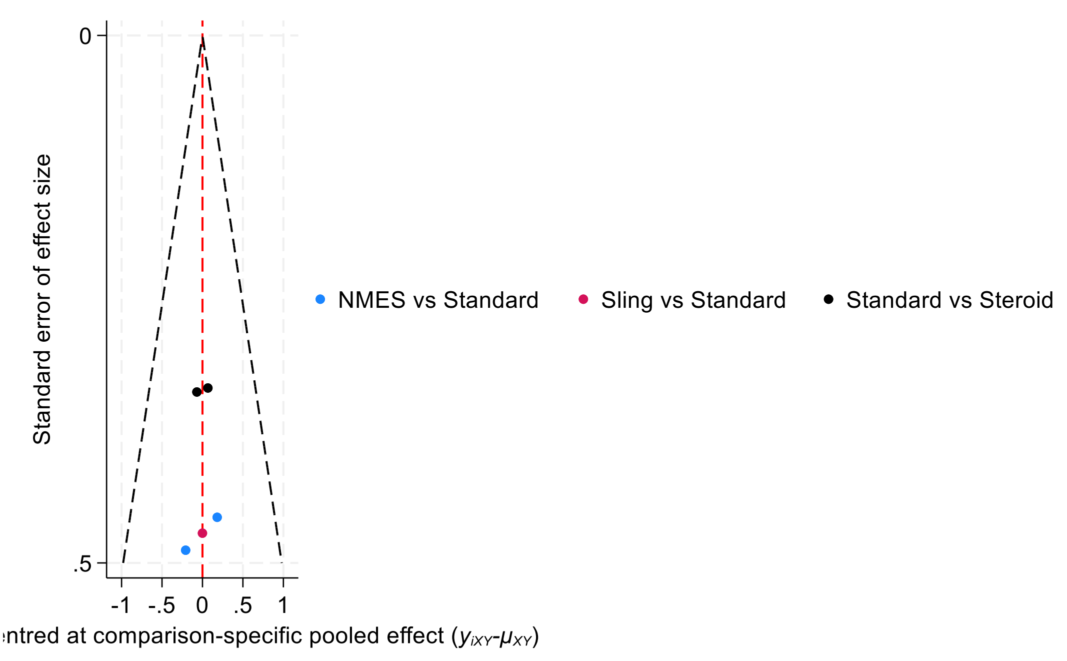

Egger's test P = 0.847

Table S4A. Results of local inconsistency tests (node-splitting analysis)

Outcome	Comparison	Coefficient			*P*
		Direct	Indirect	Difference	
Shoulder Subluxation Distance	Electroacupuncture Standard	0.1453	1.1035	-0.9583	0.974
	NMES Sling	1.0593	5.1757	-4.1164	0.977
	NMES Standard	2.2074	-0.5418	2.7492	0.962
	Standard Taping	0.6792	0.3777	0.3015	0.998
	Standard rPMS	-0.9623	-1.2635	0.3012	0.998
Pain at Rest	Electroacupuncture Standard	0.4429	0.0943	0.3487	0.990
	NMES Sling	-0.0113	0.1369	-0.1482	0.933
	NMES Standard	0.228	0.0801	0.148	0.933
	Sling Standard	0.0911	0.2395	-0.1484	0.933
	Standard Steroid	-0.7986	-1.6836	0.8849	0.995
	Standard Taping	0.2891	-0.5958	0.8849	0.995
	Standard rPMS	-0.0571	-0.942	0.8849	0.995
Pain during Activity	NMES Standard	0.2405	0.0529	0.1876	0.996
	Sling Standard	0.3173	0.1636	0.1536	0.999
	Standard Steroid	-0.8067	-1.2875	0.4807	0.997
	Standard Taping	-1.044	-1.525	0.481	0.998
	Standard rPMS	0.2071	-0.2738	0.4809	0.997
Fugl-Meyer Assessment	NMES Sling	-0.9589	-0.1027	-0.8563	0.240
	NMES Standard	0.2005	-0.6558	0.8562	0.240
	Sling Standard	0.3031	1.1594	-0.8563	0.240
	Standard Taping	1.0833	1.3114	-0.2282	0.999
PROM Shoulder Abduction	NMES Standard	1.8028	-0.0348	1.8376	0.975
	Sling Standard	-0.1043	3.7098	-3.8142	0.985
	Standard Steroid	0.6795	-2.926	3.6055	0.980

Table S4B. Global inconsistency assessment (design-by-treatment interaction model).

Outcome	χ^2	df	*P*
Shoulder Subluxation Distance	0.40	1	0.527
Pain at Rest	0.01	1	0.933
Pain during Activity	0.98	1	0.323
Fugl-Meyer Assessment	1.38	1	0.240
PROM Shoulder Abduction	8.18	1	0.004

Table S5. SUCRA Values of Treatments for Shoulder Subluxation in Stroke Patients.

	Shoulder Subluxation Distance	Pain at rest	Pain during Activity	Fugl-Meyer Assessment	Shoulder PROM
**NMES**	**84.9**	51.4	48.3	55.4	0.0
**Sling**	57.9	40.1	49.3	1.4	57.3
**Taping**	20.6	30.3	**87.7**	**98.5**	-
**Steroid**	-	**73.6**	82.5	-	**95.1**
**Electroacupuncture**	42.7	58.1	-	-	-
**rPMS**	57.9	46.2	8.3	-	-
**Standard**	35.9	40.1	23.9	44.7	47.6

Table S6. Indirectness for all studies

Author (Year)	Population	Intervention	Outcome	Comparisons	Indirectness
Anke van Bladel (2017)	Low	Low	Low	Low	Low
Canan Turkkan (2017)	Low	Low	Low	Low	Low
Chen Lavi (2022)	Low	Low	Low	Low	Low
Engin Koyuncu (2010)	Low	Low	Low	Low	Low
JongEun Yim (2024)	Low	Low	Low	Low	Low
Kenta Fujimura (2024)	Low	Low	Low	Low	Low
Lakse E (2009)	High | shoulder pain patient	Low	Low	Low	Moderate
Lin Yang (2018)	High | shoulder pain patient	Low	Low	Low	Moderate
Minghong Sui (2021)	Low	Low	Low	Low	Low
Ozgur Z. Karaahmet (2018)	Low	Low	Low	Low	Low
Pouran D. Faghri (1994)	Low	Low	Low	Low	Low
Sandra L. Linn (1999)	Low	Low	Low	Low	Low
Subhasish Chatterjee (2016)	Low	Low	Low	Low	Low

Table S7. Confidence in Network Meta-analysis (CINeMA) final report

(A) CINeMA confidence rating for Shoulder Subluxation Distance

Comparison	Number of studies	Within-study bias	Reporting bias	Indirectness	Imprecision	Heterogeneity	Incoherence	Confidence rating
Electroacupuncture:Standard	1	No concerns	Low risk	No concerns	Some concerns	Some concerns	No concerns	Moderate
NMES:Sling	2	Some concerns	Low risk	No concerns	Some concerns	Some concerns	No concerns	Low
NMES:Standard	4	Some concerns	Low risk	No concerns	No concerns	Some concerns	No concerns	Moderate
Standard:Taping	4	Some concerns	Low risk	No concerns	No concerns	Major concerns	No concerns	Low
rPMS:Standard	2	Some concerns	Low risk	No concerns	Some concerns	Some concerns	No concerns	Low
Electroacupuncture:NMES	0	No concerns	Low risk	No concerns	Some concerns	Some concerns	No concerns	Moderate
Electroacupuncture:Sling	0	Some concerns	Low risk	No concerns	Major concerns	No concerns	No concerns	Low
Electroacupuncture:Taping	0	No concerns	Low risk	No concerns	Some concerns	Some concerns	No concerns	Moderate
Electroacupuncture:rPMS	0	No concerns	Low risk	No concerns	Major concerns	No concerns	No concerns	Low
NMES:Taping	0	Some concerns	Low risk	No concerns	No concerns	Some concerns	No concerns	Moderate
NMES:rPMS	0	Some concerns	Low risk	No concerns	Some concerns	Some concerns	No concerns	Low
Sling:Standard	0	Some concerns	Low risk	No concerns	Some concerns	Some concerns	No concerns	Low
Sling:Taping	0	Some concerns	Low risk	No concerns	Some concerns	Some concerns	No concerns	Low
rPMS:Sling	0	Some concerns	Low risk	No concerns	Major concerns	No concerns	No concerns	Low
rPMS:Taping	0	Some concerns	Low risk	No concerns	Some concerns	Some concerns	No concerns	Low

(B) CINeMA confidence rating for Pain at Rest

Comparison	Number of studies	Within-study bias	Reporting bias	Indirectness	Imprecision	Heterogeneity	Incoherence	Confidence rating
Electroacupuncture:Standard	1	No concerns	Low risk	No concerns	Major concerns	No concerns	No concerns	Low
NMES:Sling	2	Some concerns	Low risk	No concerns	Major concerns	No concerns	No concerns	Low
NMES:Standard	3	Some concerns	Low risk	No concerns	Some concerns	Some concerns	No concerns	Low
Sling:Standard	1	Some concerns	Low risk	No concerns	Major concerns	No concerns	No concerns	Low
Standard:Steroid	2	Major concerns	Low risk	Some concerns	Some concerns	Some concerns	No concerns	Low
Standard:Taping	2	Some concerns	Low risk	No concerns	Major concerns	No concerns	No concerns	Low
rPMS:Standard	2	Some concerns	Low risk	No concerns	Major concerns	No concerns	No concerns	Low
Electroacupuncture:NMES	0	No concerns	Low risk	No concerns	Major concerns	No concerns	No concerns	Low
Electroacupuncture:Sling	0	No concerns	Low risk	No concerns	Major concerns	No concerns	No concerns	Low
Electroacupuncture:Steroid	0	Some concerns	Low risk	Some concerns	Major concerns	No concerns	No concerns	Low
Electroacupuncture:Taping	0	Some concerns	Low risk	No concerns	Major concerns	No concerns	No concerns	Low
Electroacupuncture:rPMS	0	Some concerns	Low risk	No concerns	Major concerns	No concerns	No concerns	Low
NMES:Steroid	0	Some concerns	Low risk	No concerns	Major concerns	No concerns	No concerns	Low
NMES:Taping	0	Some concerns	Low risk	No concerns	Major concerns	No concerns	No concerns	Low
NMES:rPMS	0	Some concerns	Low risk	No concerns	Major concerns	No concerns	No concerns	Low
Sling:Steroid	0	Some concerns	Low risk	No concerns	Major concerns	No concerns	No concerns	Low
Sling:Taping	0	Some concerns	Low risk	No concerns	Major concerns	No concerns	No concerns	Low
rPMS:Sling	0	Some concerns	Low risk	No concerns	Major concerns	No concerns	No concerns	Low
Steroid:Taping	0	Major concerns	Low risk	Some concerns	Some concerns	Some concerns	No concerns	Low
rPMS:Steroid	0	Major concerns	Low risk	Some concerns	Major concerns	No concerns	No concerns	Very low
rPMS:Taping	0	Some concerns	Low risk	No concerns	Major concerns	No concerns	No concerns	Low

(C) CINeMA confidence rating for Pain during Activity

Comparison	Number of studies	Within-study bias	Reporting bias	Indirectness	Imprecision	Heterogeneity	Incoherence	Confidence rating
NMES:Standard	3	Some concerns	Low risk	No concerns	No concerns	No concerns	No concerns	Moderate
Sling:Standard	1	Some concerns	Low risk	No concerns	No concerns	Some concerns	No concerns	Moderate
Standard:Steroid	2	Major concerns	Low risk	Some concerns	No concerns	Some concerns	No concerns	Low
Standard:Taping	1	No concerns	Low risk	Some concerns	No concerns	Some concerns	No concerns	Moderate
rPMS:Standard	2	Some concerns	Low risk	No concerns	No concerns	No concerns	No concerns	Moderate
NMES:Sling	0	Some concerns	Low risk	No concerns	No concerns	Major concerns	No concerns	Low
NMES:Steroid	0	Major concerns	Low risk	Some concerns	No concerns	Some concerns	No concerns	Low
NMES:Taping	0	Some concerns	Low risk	Some concerns	Some concerns	No concerns	No concerns	Low
NMES:rPMS	0	Some concerns	Low risk	No concerns	No concerns	Some concerns	No concerns	Moderate
Sling:Steroid	0	Major concerns	Low risk	Some concerns	Some concerns	No concerns	No concerns	Low
Sling:Taping	0	Some concerns	Low risk	Some concerns	Some concerns	Some concerns	No concerns	Low
rPMS:Sling	0	Some concerns	Low risk	No concerns	Some concerns	No concerns	No concerns	Moderate
Steroid:Taping	0	Some concerns	Low risk	Some concerns	No concerns	Major concerns	No concerns	Low
rPMS:Steroid	0	Major concerns	Low risk	Some concerns	No concerns	Some concerns	No concerns	Low
rPMS:Taping	0	Some concerns	Low risk	Some concerns	No concerns	Some concerns	No concerns	Low

(D) CINeMA confidence rating for Fugl-Meyer assessment

Comparison	Number of studies	Within-study bias	Reporting bias	Indirectness	Imprecision	Heterogeneity	Incoherence	Confidence rating
NMES:Sling	2	Some concerns	Low risk	No concerns	No concerns	Some concerns	No concerns	Moderate
NMES:Standard	1	Major concerns	Low risk	No concerns	No concerns	Major concerns	No concerns	Very low
Sling:Standard	1	Some concerns	Low risk	No concerns	No concerns	Major concerns	No concerns	Low
Standard:Taping	1	Some concerns	Low risk	No concerns	No concerns	Major concerns	No concerns	Low
NMES:Taping	0	Some concerns	Low risk	No concerns	No concerns	Major concerns	No concerns	Low
Sling:Taping	0	Some concerns	Low risk	No concerns	No concerns	Major concerns	No concerns	Low

(E) CINeMA confidence rating for shoulder PROM

Comparison	Number of studies	Within-study bias	Reporting bias	Indirectness	Imprecision	Heterogeneity	Incoherence	Confidence rating
NMES:Sling	2	Some concerns	Low risk	No concerns	No concerns	Some concerns	Major concerns	Low
Sling:Standard	1	Some concerns	Low risk	No concerns	No concerns	Major concerns	Major concerns	Low
Standard:Steroid	2	Major concerns	Low risk	Some concerns	No concerns	Some concerns	Major concerns	Low
NMES:Standard	0	Some concerns	Low risk	No concerns	No concerns	Major concerns	Major concerns	Low
NMES:Steroid	0	Some concerns	Low risk	No concerns	No concerns	Major concerns	Major concerns	Low
Sling:Steroid	0	Major concerns	Low risk	Some concerns	No concerns	Major concerns	Major concerns	Very low
